# New insights into the GINS complex explain the controversy between existing structural models

**DOI:** 10.1038/srep40188

**Published:** 2017-01-10

**Authors:** Marta Carroni, Matteo De March, Barbara Medagli, Ivet Krastanova, Ian A. Taylor, Heinz Amenitsch, Hiroyuchi Araki, Francesca M. Pisani, Ardan Patwardhan, Silvia Onesti

**Affiliations:** 1 Department of Life Sciences, Imperial College London, London, United Kingdom; 2 Structural Biology Laboratory, Elettra - Sincrotrone Trieste, Trieste 34149, Italy; 3 Mill Hill Laboratory, The Francis Crick Institute, The Ridgeway, Mill Hill, London NW7 1AA, UK; 4 Institute of Inorganic Chemistry, Graz University of Technology, Stremayerg. 6/IV, 8010 Graz, Austria; 5 Division of Microbial Genetics, National Institute of Genetics, Shizuoka, Japan; 6 Istituto di Biochimica delle Proteine, Consiglio Nazionale delle Ricerche, Napoli, Italy; 7 Protein Data Bank in Europe, European Molecular Biology Laboratory, European Bioinformatics Institute (EMBL-EBI), Wellcome Trust Genome Campus, Hinxton, Cambridge CB10 1SD, UK

## Abstract

GINS is a key component of eukaryotic replicative forks and is composed of four subunits (Sld5, Psf1, Psf2, Psf3). To explain the discrepancy between structural data from crystallography and electron microscopy (EM), we show that GINS is a compact tetramer in solution as observed in crystal structures, but also forms a double-tetrameric population, detectable by EM. This may represent an intermediate step towards the assembly of two replicative helicase complexes at origins, moving in opposite directions within the replication bubble. Reconstruction of the double-tetrameric form, combined with small-angle X-ray scattering data, allows the localisation of the B domain of the Psf1 subunit in the free GINS complex, which was not visible in previous studies and is essential for the formation of a functional replication fork.

Chromosomal DNA replication requires a large number of protein factors whose activity is tightly regulated^1^. In eukaryotic cells, replication is initiated simultaneously at multiple origins, where DNA is unwound and the replicative machinery assembled to form two replication forks that move in opposite directions^2^. The helicase core of the replicative fork is formed by the CMG complex, which includes the MCM2–7 helicase, Cdc45 and the GINS complex^3^. The MCM complex is loaded onto origins as an inactive helicase and is activated upon Cdc45 and GINS binding^4^,^5^.

GINS is a 100 kDa complex comprising four polypeptide chains (Sld5, Psf1, Psf2 and Psf3) all essential for DNA replication^6^,^7^,^8^. Beside its role as a key component of the CMG complex, GINS mediates a large number of interactions with many replication factors^9^,^10^.

Three crystal structures of the heterotetrameric human GINS complex (hGINS) were independently determined^11^,^12^,^13^. All these structures show the same trapezoid shape with the four subunits forming a tight bundle, held by extensive inter-subunit contacts mediated by hydrophobic interactions (Fig. 1a). The four subunits are structurally related and are likely to derive from a single protein through gene duplication and subsequent domain swap. They share a common fold made up of two regions: the α-helical A domain and the smaller β rich B domain. Sld5 and Psf1 possess the A domain at the N terminus and the B domain at the C terminus, while in Psf2 and Psf3 the two regions are swapped (Fig. 1b). The B domain of Psf1 is sensitive to proteolysis, suggesting that it is connected to the core of the complex via a flexible linker. Two of the crystal structures^11^,^12^ were determined using a Psf1ΔB mutant, whereas no electron density could be seen for the B domain when the full-length protein was crystallized^13^.

Two types of archaeal homologues have been identified, one similar to Sld5/Psf1 (called Gins51) and another (Gins23) closer to Psf2/3^14^,^15^. Only a subset of archaea possess two GINS subunits, whereas the majority of Euryarchaeota have a single subunit, more similar to Gins51, thus indicating that all GINS protein diverged from a common ancestor. The crystal structure of the archaeal GINS complex from *T. kodakaraensis* shows a tetramer composed of two copies of Gins51 and two copies of Gins23 arranged in a similar way to the human GINS complex^16^, suggesting that the functional form of the complex is essentially conserved from archaea to eukaryotes.

Surprisingly, the crystal structures differ significantly from the images of the isolated GINS complex taken by electron microscopy. Early studies, performed on the *Xenopus* orthologue using rotary shadowing techniques, revealed either ring-like or C shaped molecules^7^. Similar results were obtained when the same experiment was repeated with the human GINS complex^11^. A 33 Å resolution EM three-dimensional map of human GINS shows a C-shaped molecule (Fig. 1c), very different from the compact crystallographic model^17^. Various explanations have been suggested to resolve the remarkable discrepancy between the EM 3D reconstruction and the crystal structures: the presence of a small indentation in the middle of the complex that could give rise to a less electron-dense region appearing like a hole^11^; the partial disordering of the Psf3 N-terminal domain creating a pore^13^; a large conformational change involving the opening up of the four subunits^17^ (Fig. 1d). The first two explanations fail to reconcile the size and shape of the crystallographic structure with that of the EM 3D reconstruction, while a large conformational change that completely unfolds the four-subunit bundle requires the disruption of the tight interaction and extended interface seen by protein crystallography. It has to be stressed that the size of GINS (100 kDa) places it at (or below) the limit of single particle analysis by EM.

In summary, the discrepancies between the crystallographic and EM structures of the free GINS complex are still unanswered. Here, we show that samples of recombinant human GINS comprise a small population consisting of dimers of tetramers and that this is the fraction that is more amenable to be visualised and analysed by EM. We present a new low resolution three-dimensional EM reconstruction of the hGINS complex, which confirms the formation of double tetramers. We report small angle X-ray scattering (SAXS) studies showing that hGINS is mainly a single tetramer in solution with a compact shape, in good agreement with the crystallographic data, with a protrusion that is likely to correspond to the Psf1 B domain, which is flexible in the free complex. Our results provide an explanation for the differences observed in the structural studies performed to date and suggests that, even though the free GINS complex is mainly a single tetramer in solution, a small double-tetrameric population can be visualised. GINS double tetramers may be recruited to double-hexameric MCM complexes at replication origins^18^,^19^,^20^, to form two active helicases with opposite polarities.

## Results

### The human GINS complex can form dimers of tetramers in solution

Recombinant hGINS proteins Sld5, Psf1, Psf2 and Psf3 were co-expressed in *E. coli* cells and purified via Ni-affinity chromatography followed by heparin and size-exclusion chromatography. During size-exclusion chromatography, the hGINS complex was eluted as a single major peak corresponding to an apparent molecular weight (MW) of 100 kDa, in accordance with the size of the hetero-tetrameric assembly. In addition, a small fraction of the complex dimerised, as indicated by the migration of a second peak at a position corresponding to the MW of two tetramers (approximately 200 kDa, Fig. 2). The presence of this second peak was not dependent on the buffer used or on the presence of the hexa-histidine tag; subsequent size-exclusion chromatography runs of the monomeric peak fractions indicated that the two forms of the complex are in slow equilibrium in solution (Fig. 2a). In order to assess the exact size of this putative double tetramer, size-exclusion chromatography coupled with multi-angle laser light scattering (SEC-MALLS) was performed, showing two peaks with MW of 95 kDa and 190 kDa, with a ratio of 10:1 of tetramer to double tetramer (Fig. 2b). These results show that a fraction of the hGINS complex dimerises in solution.

Gel-filtration fractions containing the two species were analysed by negative stain electron microscopy and the resulting micrographs showed clear differences (Fig. 2c). The low MW fraction appeared as small white spots, while larger globular particles were visible in the high MW peak. It has to be noted that the single GINS tetramer is at the size-limits of EM analysis. Moreover, a similar behaviour was observed when the yeast GINS complex was visualised by EM after size exclusion chromatography (Supplementary Figure S1) showing that this dimerisation is not a peculiarity of the human complex.

Taken together, these findings suggest that the GINS complex forms dimers of tetramers in solution and that, even though the single tetramer is the predominant form, double tetramers can be detected and visualised by EM more easily.

### EM reconstruction of the human GINS complex confirms the existence of dimers of tetramers

To explain the structural differences observed to date, we have performed a negative-stain EM reconstruction of human GINS, using single particle analysis. Negative-stain images were collected and out of 20,000 automatically picked particles, around 12,000 were selected based on alignment and multivariate statistical analysis (MSA). During the initial rounds of classification, it was found that the presence of a large number of smaller circular particles, with a diameter of ~50 Å, caused problems during the alignment, resulting in the generation of featureless class-averages. These small particles were removed, as they did not add details to the final reconstruction (Supplementary Figure S2). Similar observations of small particles on the background have been previously reported when imaging the *X. laevis* complex^7^.

We generated a 3D structure of the human GINS complex using around 8000 particles (Supplementary Figure S2). At the sigma threshold that allows to distinguish clearly the molecule density from the noise, the reconstruction corresponds to a molecular weight of ~200 kDa. At higher sigma threshold, corresponding to a MW of ~100 kDa, two high-density cores are visible. These observations suggested that our reconstructed EM structure represents a dimer of GINS tetramers. As a comparison, the EM map of the GINS complex described in Boskovic *et al*.^17^, EMD 1355) is displayed to the threshold that reproduces the published images. The volume enclosed by this map is roughly 170 kDa, suggesting that also the published map might represent a dimer of GINS tetramers.

Based on these observations and the biochemical evidence, we refined our EM reconstruction by applying 2-fold symmetry. Eigen-image analysis further justified the use of C2 symmetry (Fig. 3a). The C2 hGINS map, at 30 Å resolution (Supplementary Figure S3), resembles an elongated trapezoid with two small protrusions sticking out from each of the main core densities (Fig. 3b). Contrary to previous EM reports, our reconstruction does not possess a central pore. However, a groove is present between the two GINS tetramers, which appears as a hole when the map is displayed at high sigma threshold (Supplementary Figure S2). The defocus at which images are taken as well as the negative stain procedure itself can emphasise the appearance of holes in a single molecule, but this artefact is minimized in the averaging process of images taken at different defoci.

A dimer of tetramers found in one of the published crystal structures (PDB ID: 2EHO^12^) can be automatically fitted as a rigid body in our EM reconstruction using Chimera^21^ with a cross correlation coefficient (CCC) of 0.88 (Fig. 3b). The interface between the two tetramers includes a number of residues that are conserved in all vertebrate sequences, although not throughout all eukaryotic species. However, the observation of a similar behaviour across yeast and human species suggests this is a conserved feature of the GINS complex.

Interestingly, the coordinates from the crystal structure do not account for the small protruding densities. As these lie in proximity of the last modelled residue of the Psf1 subunits, it is reasonable to speculate that they represent the Psf1 B domains which are easily removed by proteases in the free GINS tetramer.

### SAXS data indicates the presence of a compact GINS tetramer in solution and confirms the position of the Psf1 B domain

SAXS data were collected to further characterise the human GINS complex in solution and assess possible conformational changes. The final model, obtained from the experimental scattering curve after data processing and P(r) calculation, has structural parameters R_g_ = 33.7 Å and D_max_ = 110 Å and shows a compact parallelepiped with a lateral protrusion (Fig. 4a,b). The main core is in good agreement with the crystallographic tetrameric structure, which can be automatically fitted by Chimera with a CCC of 0.92. These data confirm that in solution the major GINS population corresponds to a tetramer with a shape consistent with the crystal structures^11^,^12^,^13^. The EM structure of the CMG complex also confirms that the GINS crystallographic tetramer can also easily fit into the CMG density without conformational changes^4^,^5^.

The lateral protrusion, consistently found in all *ab initio* 30 models, lies in proximity of the last visible Psf1 residues of the crystal structure of the isolated GINS complex. Within the context of the CMG^5^ the Psf1 B domain packs against Cdc45, but biochemical data consistently indicate that in the free GINS complex this domain is connected to the GINS core by a linker prone to proteolysis, suggesting that the domain is flexible and exposed to the solvent. Therefore the missing domain for Pfs1 was modelled by fitting it into the lateral protrusion (Fig. 4b). This hybrid model (GINS crystal structure + modelled Psf1 B domain in extended conformation) was used to generate a simulated SAXS curve, that fitted the experimental data better than the crystal structure lacking the Psf1 B domain (PDB ID: 2EHO) or the full-length GINS complex derived from the 4.7 Å EM MAP (PDB ID: 3JC5), Fig. 4c). We collected further SAXS data from a deletion mutant lacking the B-domain (Psf1ΔB) and confirmed that the structure is more compact (R_g_ = 29.39 Å and D_max_ = 100 Å) and the data show a better fit when the GINS crystal structure was used, without the modelled Psf1 B domain (Supplementary Figure S4).

When two SAXS maps of a single tetramer are superimposed to the EM map for the double tetramer, the overall shape is very similar and the Psf1 B domains positions from SAXS are in close proximity to the protrusions of the EM electron density (Fig. 4d).

## Discussion

**Figure 1:**
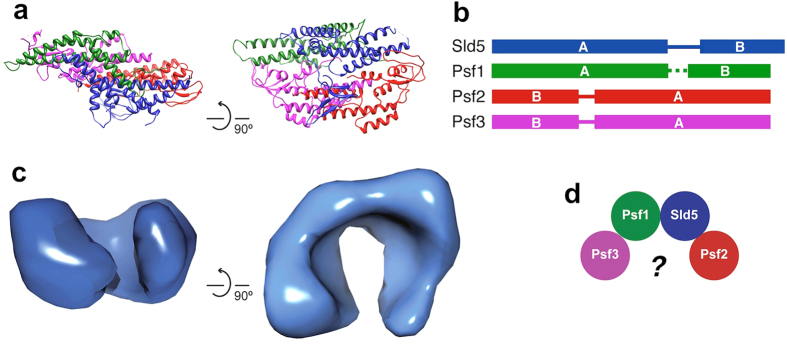
Structure and architecture of GINS complex. **(a)** Ribbon representation of the crystallographic structure of human GINS^12^ (PDB ID: 2EHO) **(b)** Schematic diagram of the GINS subunits, highlighting the domain swap between the α-helical (A) and the β-rich (B) domains. **(c)** EM map of hGINS^17^ (EMD-1355) showing a C-shaped molecule. **(d)** To resolve the discrepancy between crystallographic and EM data a large rearrangement of the four GINS subunits has been proposed^17^.

Despite the fact that structural data have long been available for the human free GINS complex, there was a remarkable inconsistency between the information from crystallography and electron microscopy (Fig. 1): whereas all the crystal structures^11^,^12^,^13^,^16^ are compact trapezoids, EM data^7^,^11^,^17^ show the presence of a partially open ring, raising the issue of the actual shape of the complex in solution. The explanations provided so far to resolve the discrepancy have not been satisfactory. Whereas it is true that the tetrameric ring complex has a low-density region in the middle that can appear as a hole in negative stain EM^11^, the size and shape of the crystal and EM structures are different. A similar observation can be done for the hypothesis that the hole results from the “unplugging” of the Psf3 N-terminal domain^13^, adding to the fact that no evidence of such conformational change has ever been presented. The most dramatic explanation required a large conformational change to open up the four GINS subunits from their compact crystallographic assembly to form a C-shaped structure and the disruption of a large network of hydrophobic interactions with huge energetic costs^9^.

We have addressed the problem by collecting new low resolution electron microscopy data from both the human and yeast GINS complexes, and carrying out a 3D reconstruction from negative-stain micrographs of the recombinant human sample. Our reconstruction can be interpreted as a dimeric complex with two GINS tetramers related by a 2-fold axes (Fig. 3). This new EM map does not show a central pore, although a groove is present between the two GINS tetramers: when the map is displayed at high sigma threshold this groove can appear as a “hole” (Supplementary Figure S2). Due to the low resolution of the EM map, we cannot say with absolute confidence which is the exact configuration of the dimer; to be conservative, we choose to fit into the map a crystallographic dimer, which provides a good match.

**Figure 2:**
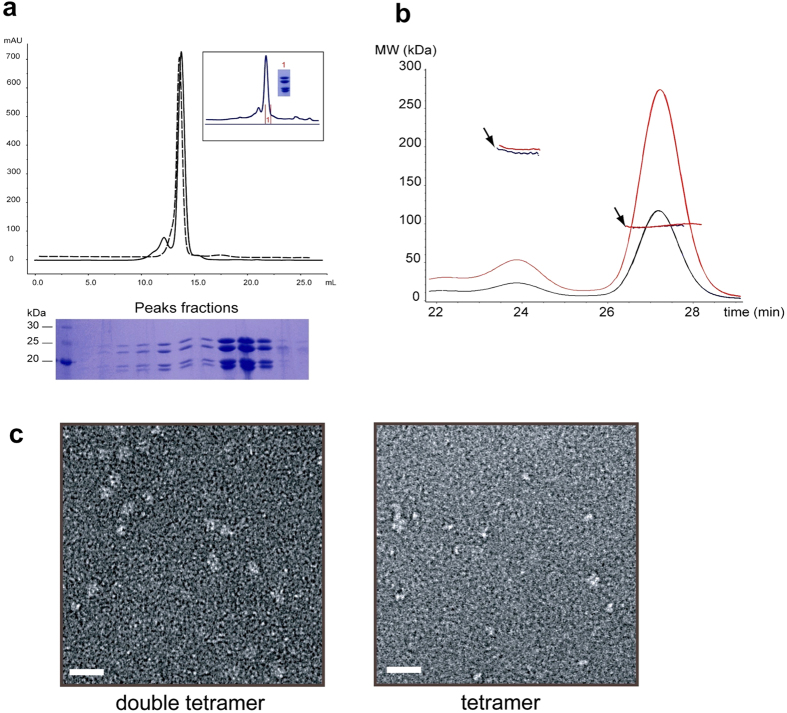
Dimerisation of hGINS. **(a)** Size-exclusion chromatography profile of a monomeric peak fraction run minutes (dashed line) or days (solid line) after elution. In the inset is indicated the fraction that was run again (labelled 1 in red) and the corresponding Coomassie-stained SDS gel of the sample. Coomassie-stained 12% SDS-polyacrylamide gel show the four subunits eluting from the peaks. **(b)** SEC-MALLS analysis of hGINS complex. Elution profiles generated by applying 100 μL of 5 (black line) and 10 mg/mL (red line) hGINS onto a Superdex 200 column coupled with differential refractive index detection are shown. Arrows indicate the MW measurements of the species eluted in the two peaks. **(c)** Samples from the two size exclusion chromatography peaks were stained and visualised by EM. Scale bar is 200 Å.

**Figure 3:**
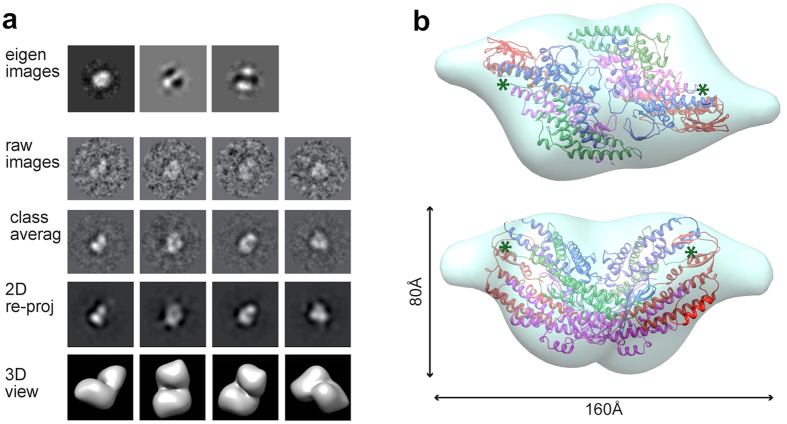
Three dimensional reconstruction of the hGINS double tetramer. **(a)** Outline of the 3D reconstruction procedure. Eigen-images indicate the presence of 2-fold symmetry. **(b)** Double tetramer EM map with a fitted crystallographic dimer^12^ (PDB ID: 2EHO, chains E to L). The green asterisks indicate the location of the last residue of Psf1.

**Figure 4:**
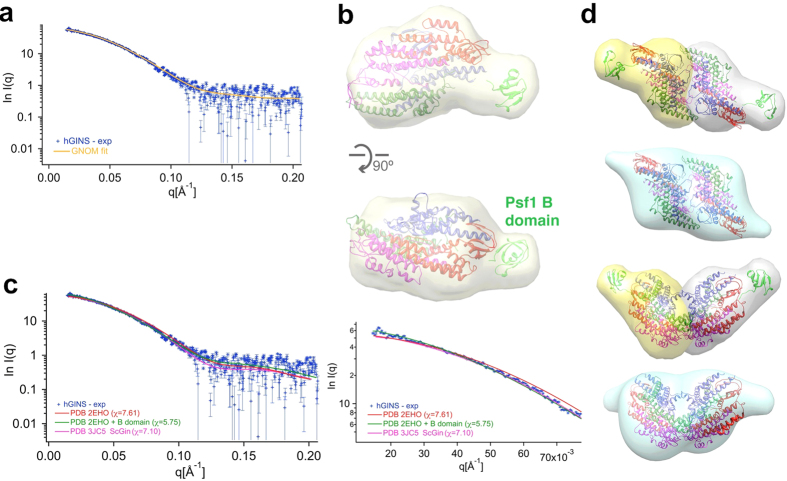
SAXS analysis of the full-length hGINS complex. **(a)** Experimental SAXS profile (blue crosses) and GNOM fit (yellow line). **(b)** Final model reconstructed from the scattering curve. The crystal structure of a GINS tetramer^12^ (PDB ID: 2EHO) was fitted onto the SAXS map; to illustrate the putative position of the Psf1 B domain, the equivalent region from Sld5 from has been fitted into the protrusion (in green). **(c)** The simulated SAXS curves from the crystallographic GINS structure (red), from a model including the exposed Psf1 B domain (green) and from the full-length GINS complex derived from the 4.7 Å EM MAP (PDB ID: 3JC5, magenta), are compared with the experimental SAXS data. An inset shows the fit at low angles. **(d)** Agreement between SAXS and EM. Two SAXS envelope (in grey and yellow, respectively) were overlapped with the EM double tetramer and compared with the EM double tetrameric map.

In agreement, both size exclusion chromatography and SEC-MALLS analysis show the presence of a small population of dimers of tetramers (Fig. 2). SAXS data confirm that the dominant population in solution is the tetrameric form and can be fitted easily by the crystallographic structures, without any conformational change (Fig. 4). However, this tetrameric population is poorly detected by EM methods due to the small size (Fig. 2 and Supplementary Figure S1).

Both the EM and the SAXS model show electron density protrusions that are not accounted for by the crystallographic model, and are in close proximity to the last ordered residue of Psf1 in the free GINS complex. The Psf1 B domain has bee shown to be essential in DNA replication and CMG assembly^11^,^22^. Within the CMG complex, the Psf1 B domain is adjacent to Cdc45, but in solution is prone to proteolysis, suggesting a more exposed location. Recent biochemical data suggests that the B domain interacts with the Dpb2 subunit of polymerase ε^23^ and the interaction is critical to link the helicase to the leading strand polymerase during the progression of the replication fork. This requires an exposed location for the Psf1 B domain, suggesting the possibility that a conformational change may also occur within the CMG complex.

Data from both the archaeal and the yeast system indicates that, after an initial association step^24^,^25^ the MCM complex loads onto DNA as double hexamer^18^,^19^,^20^,^26^. A head-to-head double hexamer, which separates into two single hexamers with opposite orientation, is indeed a useful tool to provide two helicase engines moving in opposite direction along the two arms of the replication bubble. Consequently, a double-hexameric MCM at each replication origin needs to recruit two GINS tetramers and two Cdc45 in order to assemble two CMG complexes moving in opposite directions. The conservation of a double-tetrameric GINS complex population from yeast to human suggests a functional purpose. This double tetramer may be the form recruited by MCM at origins and thus represent an intermediate step towards the assembly of two active helicases with opposite polarities.

## Methods

### Protein purification and characterization

Recombinant human GINS with a cleavable histidine tag at the N-terminus of Psf3 was produced and purified via Ni2+ affinity chromatography following the protocol previously described^27^. An additional purification step through a heparin column was added prior to the final size exclusion chromatography. The four subunits co-eluted during all purification steps regardless of the ionic strength (up to 500 mM NaCl plus 500 mM imidazole) and pH (6.0–8.0). The Psf1ΔB mutant of the hGINS was generated by engineering a stop codon after residue 149 and purified as the full-length protein. Two duet constructs to co-express the *S. cerevisiae* GINS complex were constructed and the protein was purified by Ni^2+^ affinity followed by anionic exchange and size exclusion chromatography. The molecular mass for the human GINS complex was determined by SEC-MALLS (Further details in Supplementary Information).

### EM data collection and processing

Images of negative stain hGINS were taken on a Philips CM200 microscope at a nominal defocus range of 3 to 1 μm and at a magnification of 50,000×, resulting in a pixel size of 2 Å/pixel. Frames were CTF corrected, binned by a factor of 2 and band-pass filtered. After normalisation, ~20,000 particles were automatically picked, centered and classified based on multivariate statistical analysis (MSA). Good classes were used as references for further cycles of alignment and classification. Various reference-free models were obtained by angular reconstitution and among those in good agreement with each other the one with the lowest Euler error was used for refinement. As eigen-image analysis indicate the presence of two-fold symmetry, the asymmetric map was refined further by applying C2 symmetry. Details are presented in the Supplementary Information.

### SAXS data collection and analysis

Scattering data were collected at the Elettra SAXS beamline on a Mar300 Image Plate detector for the full length hGINS and a complex lacking the Psf1 B domain (hGINS-ΔB), at multiple concentrations (1–4 mg/ml and 0.67–5 mg/ml, respectively), at a sample-to-detector distance corresponding to a range of the scattering vector q of 0.013–0.21 Å^−1^ and 0.023–0.2 Å^−1^, respectively. Multiple exposures were collected from each sample, checked for signs of radiation damage, background subtracted and averaged. The radius of gyration and maximum dimension were calculated for both samples using GNOM^28^. Low-resolution envelopes were obtained using the ATSAS suite^29^. Additional SAXS data were collected using the in-line Size Exclusion Chromatography coupled SAXS at the B21 beamline (DIAMOND, UK). Further details are presented in Supplementary Information and Supplementary Figure S5.

## Additional Information

**How to cite this article**: Carroni, M. *et al*. New insights into the GINS complex explain the controversy between existing structural models. *Sci. Rep.* **7**, 40188; doi: 10.1038/srep40188 (2017).

**Publisher's note:** Springer Nature remains neutral with regard to jurisdictional claims in published maps and institutional affiliations.

## Supplementary Material

Supplementary Information
