# Supplementary material for: New insights into the GINS complex explain the controversy between existing structural models
Source: Sci Rep. 2017 Jan 10;7:40188. doi: 10.1038/srep40188 (PMC5223209; doi:10.1038/srep40188)
Supplement: Supplementary Information [file srep40188-s1.pdf]

**New insights into the GINS complex explain the controversy between existing structural models.**

# Supplementary Information

## Molecular mass determination by SEC-MALLS

Molecular masses of the human GINS complex were determined using on-line MALLS (multi-angle laser light scattering) coupled with size-exclusion chromatography (SEC-MALLS). A sample volume of 100 µL, at different concentrations, was applied onto a Superdex 200 10/300 GL column equilibrated in 20 mM TrisHCl pH 7.8, 150 mM NaCl, 0.5 mM TCEP (tris-2carboxyethyl phosphine) and 3 mM NaN3), at a flow rate of 0.5 mL/min. The column was mounted on a *Jasco* HPLC controlled by the Chrompass software package. As the sample was eluted from the column, the scattered light intensity of the eluate was recorded at 16 angles using a DAWN-HELEOS laser photometer (*Wyatt Technology*). The refractive index of the solvent and the specific refractive index of the dissolved protein were recorded using an OPTILAB-rEX differential refractometer (*Wyatt Technology*). The refractive index change was used to calculate the protein concentration (dn/dc 0.186 mL/g). The wavelength of the light generated by the laser photometer and the differential refractometer was at a wavelength of 658 nm. The averaged molecular mass was determined by using the software ASTRA version 5.1 (*Wyatt Technology*).

## EM data collection and processing

Negative stain data were automatically collected on a Philips CM200 operated at 200 kV, at low dose conditions, and images were taken at a nominal defocus range of -3 to -1 µm and at a magnification of 50,000× (resulting in a pixel size of 2 Å/pixel), using a CCD camera (4k×4k *TVIPS*). CCD frames were CTF corrected using the FINDCTF2D and FLIPCTF2D program (Tigris), binned by a factor of 2 and band-pass filtered between 10 and 150 Å. After normalisation, particles were automatically picked, based on variance, using the PICK-M-ALL program from the IMAGIC package (van Heel et al., 1996). Around ~20,000 particles were framed into boxes of 80 × 80 pixels. Images were first centred to their rotationally averaged total sum and then analysed by multivariate statistical analysis (MSA) and classification using IMAGIC. All further cycles of alignment were performed using the program TIMALIGN (currently BRUTEALIGN in Tigris), which uses a brute-force approach. Euler angles to class averages were assigned by angular reconstitution as implemented in IMAGIC and the first models were obtained using the C1 STARTUP procedure. This way, the use of a starting reference model was avoided. First, various reference-free models were constructed. Three-dimensional models that appeared to be in good agreement with each other, as similar angles were assigned to similar views of the particle, were considered reliable starting 3D. The one among them with lowest overall Euler error was used for refinement to obtain the asymmetric reconstruction. Eigen-images analysis revealed the presence of two-fold symmetry that was used to further refine the map through subsequent cycles of alignment, classification and angular reconstitution (van Heel et al., 2000).

## SAXS data collection and analysis

The right side of the monomeric peaks of hGINS and hGINS-ΔB from gel filtration were buffer-exchanged in 20mM Tris/HCl pH 7.4, 50mM NaCl, 5% glycerol and 5mM DTT, to obtain a range of concentrations (1, 2, 4 mg/mL for hGINS and 0.67, 1.3, 2.7, 5 mg/mL for hGINS-ΔB). SAXS data were collected at the Austrian SAXS beamline at Elettra (Amenitsch et al., 1998) equipped with a Mar300 Image Plate detector, with exposure time of 1 minute/image, at a wavelength of 1.54 Å and a sample-to-detector distance of 1775 and 1609 mm, respectively. Multiple exposures were taken for each sample, and frames were carefully examined for X-ray damage. After calibration with silver behenate, raw data were radially averaged using Fit2D. The scattering contributions of empty capillaries, water and buffers were subtracted from the total scattering intensity of all protein curves using IgorPro (http://www.wavemetrics.com). All scattering curves collected for the same sample were averaged. Data were further normalized taking into account the transmission and the detector parameters. Guinier's fit for both experimental datasets was used to check for particle aggregation and radiation damage and the scattering curves were consequently merged to obtain an artifact-free profile over the entire range. Due to a slight sample aggregation few initial points were removed both from the final hGINS and hGINS-ΔB experimental data. The absolute intensity was scaled using the scattering of BSA as reference. The experimental Rg in the Guinier's range ($q_{max}$<1.3/Rg) and the forward scattering intensity I(0) for both proteins were evaluated using IgorPro and ATSAS suite (Svergun, 2007) (Supplementary Figure 4). The experimental Rg were confirmed looking at the theoretical Rg calculated by CRYSOL from the crystal structure with PDB ID: 2EHO. The best P(r) functions were calculated by GNOM (Svergun, 1992) within a range of $q>\Pi/D_{max}$ and using $D_{max}$ of 110Å for hGINS and 100Å for hGINS-ΔB (Supplementary Figure S4). 30 successfully *ab-initio* reconstructions using DAMMIF (Franke and Svergun, 2009) were averaged and superimposed with DAMAVER/SUPCOMB (Kozin and Svergun, 2001; Volkov and Svergun, 2003) obtaining the best hGINS envelope (NSD=0.703). The validity of this model was confirmed by averaging 10 DAMMIN (Svergun, 1999) reconstructions with NSD of 0.81. The final hGINS-ΔB model was obtained by averaging 10 DAMMIN runs (NSD 0.79, Supplementary Figure S4) and it was double-validated using 10 DAMMIF reconstructions (NSD 0.75) from the experimental scattering curve and using a single DAMMIN model from the CRYSOL curve of the crystal structure (PDB ID: 2EHO). The fits of the crystal structure (PDB ID: 2EHO) and of its model with the Psf1B-domain into the 3D envelopes were done using Chimera, while FoXS (Schneidman-Duhovny et al., 2010) was used to fit the theoretical SAXS profiles on the experimental data. The X-values of each fit are reported in Figure 4 and in Supplementary Figure S4. OLIGOMER (Konarev at al., 2003) was used to exclude the presence of dimer of tetramers in solution in both hGINS and hGINS-ΔB (Supplementary Figure S4): the Rg calculated from the experimental data with OLIGOMER (33.6 Å for hGINS and 29.6 Å for hGINS-ΔB) have been compared to the theoretical Rg calculated from the crystal structure (28.7 Å) and the total fraction of monomer has been calculated as 100%. OLIGOMER has been used as a valid computational approach to study particle oligomerization (Møller at. al, 2013).

In-line Size Exclusion Chromatography (SEC) coupled SAXS data were further collected at B21 beamline (DIAMOND) through a temperature-controlled capillary from 96 well plates, mounted by the automated bioSAXS robot, within a range of scattering vector q between 0.004 $Å^{-1}$ and 0.4 $Å^{-1}$. Data processing has been carried out as described above and clearly confirmed the SAXS data presented in the paper: the SEC 100kDa peak corresponds to a single tetramer in solution with Rg=33.3 Å and Dmax=110 Å and shows a lateral protrusion associated with the presence of the flexible Psf1 domain (Supplementary Figure S5).

### Fitting and Visualization

Atomic structures were fitted into the corresponding EM and SAXS maps using the program Chimera (Pettersen et al., 2004), which was also used for the production of molecular graphics images.

## References


Amenitsch, H.A. et al. First performance assessment of the SAXS beamline at ELETTRA. *J. Synchrotron Rad.* **5**, 506-508 (1998).

Franke, D. & Svergun, D.I. DAMMIF, a program for rapid ab-initio shape determination in small-angle scattering. *J. App. Cryst.* **42**, 342-346 (2009).

Kozin, M.B. & Svergun, D.I. Automated matching of high- and low- resolution structural models. *J. App. Cryst.* **34**, 33-41 (2001).

Pettersen, E.F. et al. UCSF Chimera, a visualization system for exploratory research and analysis. *J. Comp. Chem.* **25**, 1605-1612 (2004).

Schneidman-Duhovny, D., Hammel, M. & Sali, A. FoXS: a web server for rapid computation and fitting of SAXS profiles. *Nucl. Ac. Res.* **38**, W540-544 (2010).

Svergun, D.I. Small-angle scattering studies of macromolecular solutions. *J. App. Cryst.* **40**, s10-s17 (2007).

Svergun, D.I. Determination of the regularization parameter in indirect-transform methods using perceptual criteria. *J. App. Cryst.* **25**, 495-503 (1992).

Svergun, D.I. Restoring low resolution structure of biological macromolecules from solution scattering using simulated annealing. *Biophys. J.* **76**, 2879-2886 (1999).

van Heel, M. et al. Single-particle electron cryo-microscopy: towards atomic resolution. *Quart. Rev. Biophysics* **33**, 307-369 (2000).

van Heel, M., Harauz, G., Orlova, E.V., Schmidt, R. & Schatz, M. A new generation of the IMAGIC image processing system. *J. Struct. Biol.* **116**, 17-24 (1996).

Volkov, V.V. & Svergun, D.I. (2003). Uniqueness of ab initio shape determination in small-angle scattering. *J. App. Cryst.* **36**, 860-864.

## Supplementary figures

### Supplementary Figure S1.

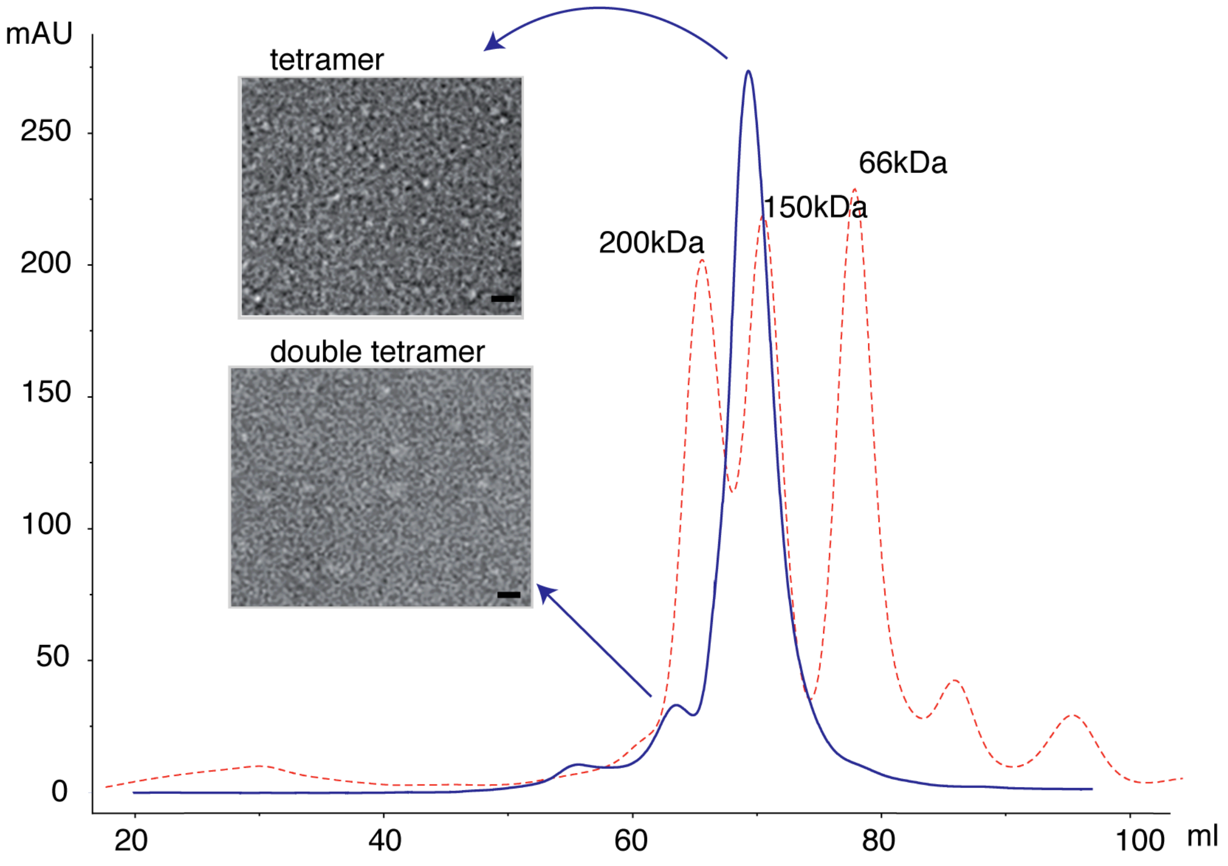


**Dimerisation of the *S. cerevisiae* GINS complexes.** The blue trace shows the elution profile of yeast GINS from a 120 mL CV Superdex 200 gel-filtration column that was calibrated using β amylase (200 kDa), alcohol dehydrogenase (150 kDa) and BSA (66 kDa) (red dashed line). Peak fractions were stained and visualised by EM and representative micrographs are shown. The main peak sample appears as a population of small white spots, while in the dimeric peak sample was possible to distinguish more defined globular molecules, similar in size to the one observed in the micrographs of human GINS. Scale bar is 100 Å.

**Supplementary Figure S2.**

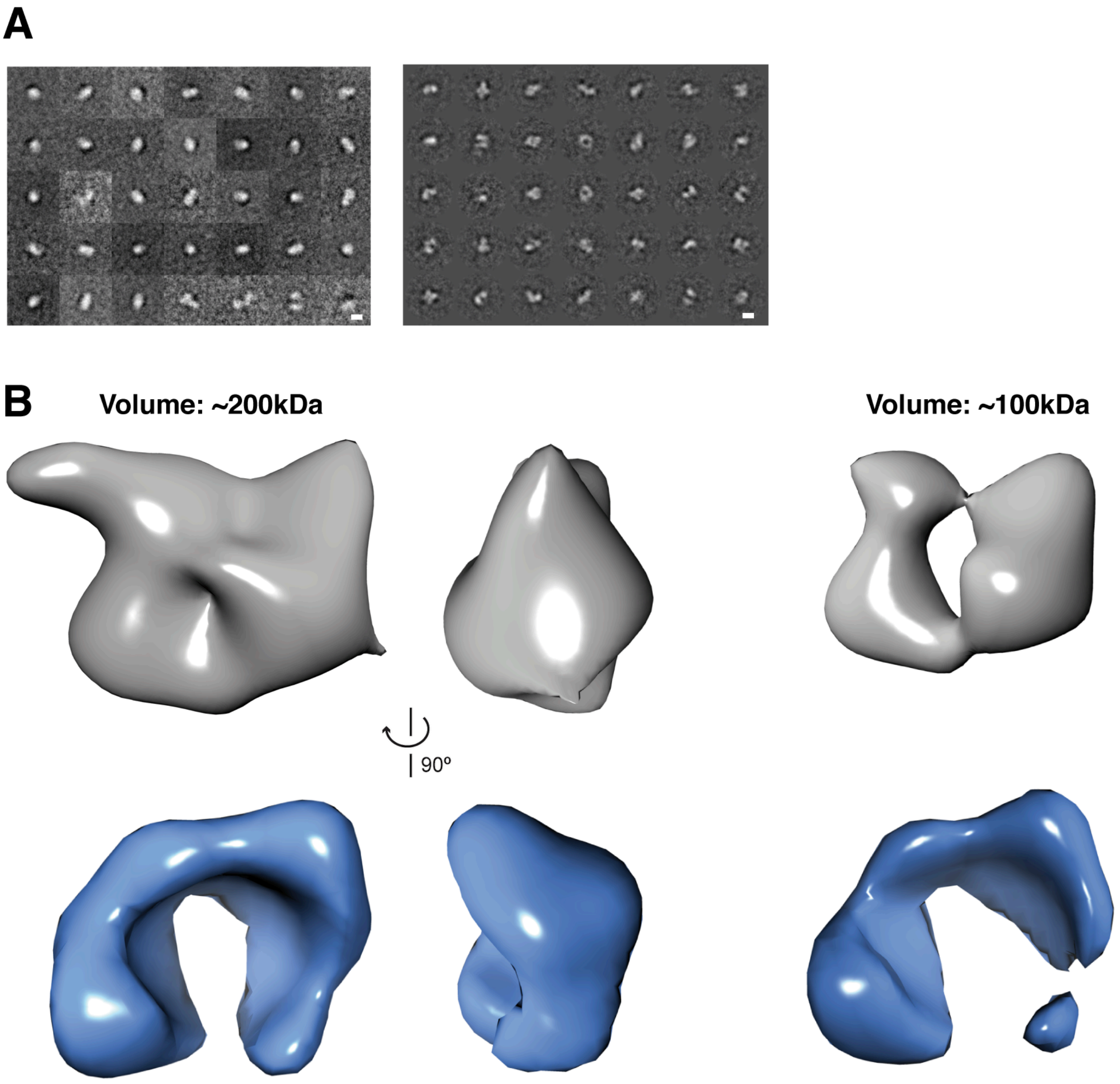


**Asymmetric reconstruction of human GINS. A.** Class-averages obtained after MSA classification of the initial dataset (left panel). A strong circular density is present in the middle of most class-averages, probably due to a large number of small round particles in the dataset. Class-averages obtained after the removal of the smaller particles from the dataset (right panel). Scale bar is 100 Å. **B.** Comparison between the initial EM map obtained in this study (grey surface) and the one deposited in the EM databank (blue surface; EMD-1355), at a threshold enclosing 200 and 100kDa, respectively.

## Supplementary Figure S3.

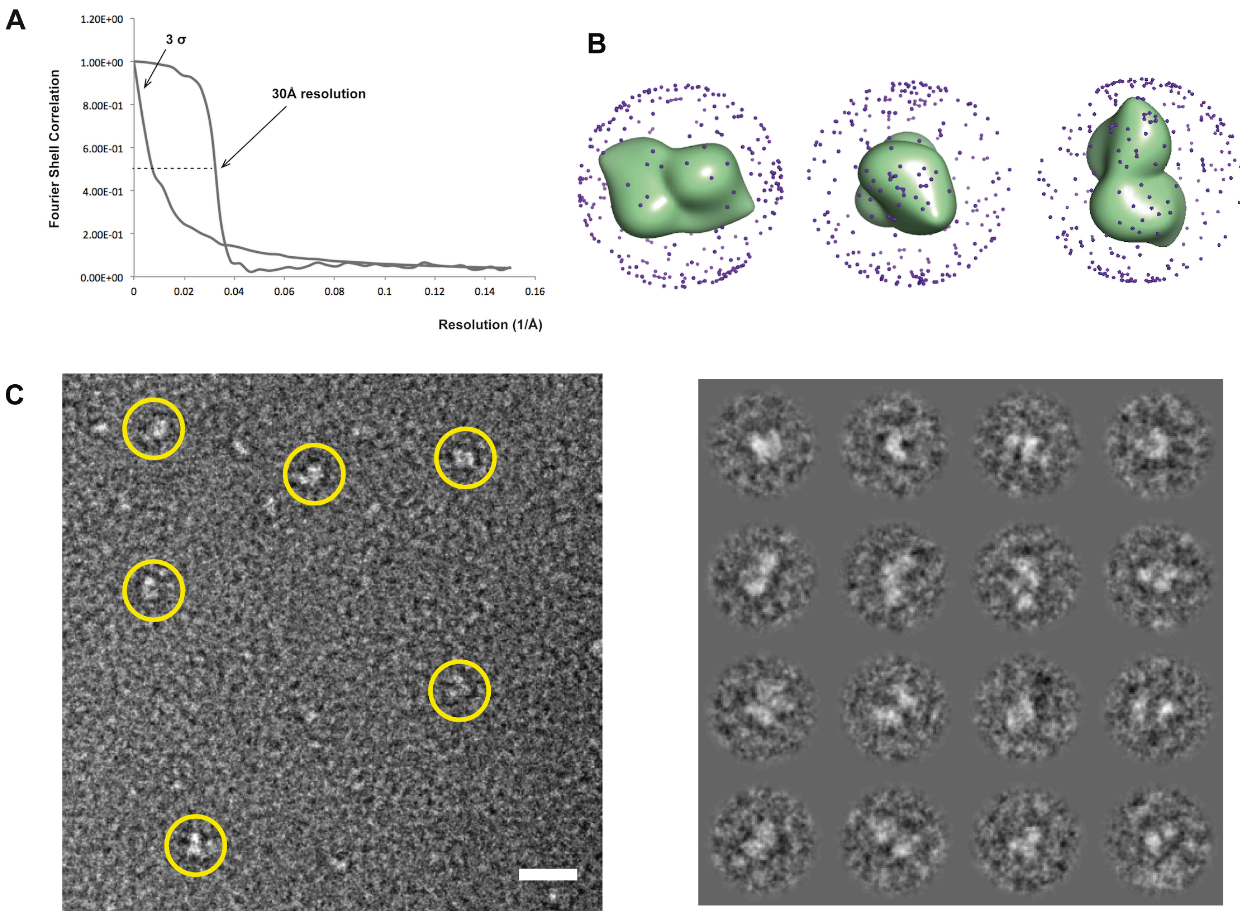


**EM analysis of the hGINS complex. A.** Resolution assessment by Fourier Shell Correlation. **B.** Distribution in the Euler sphere of the class averages used for 3D reconstruction. **C.** Representative electron micrograph of negative stain hGINS sample (left panel). Picked particles are circled in yellow. Examples of boxed single particles used for the three dimensional reconstruction (right panel).

## Supplementary Figure S4.

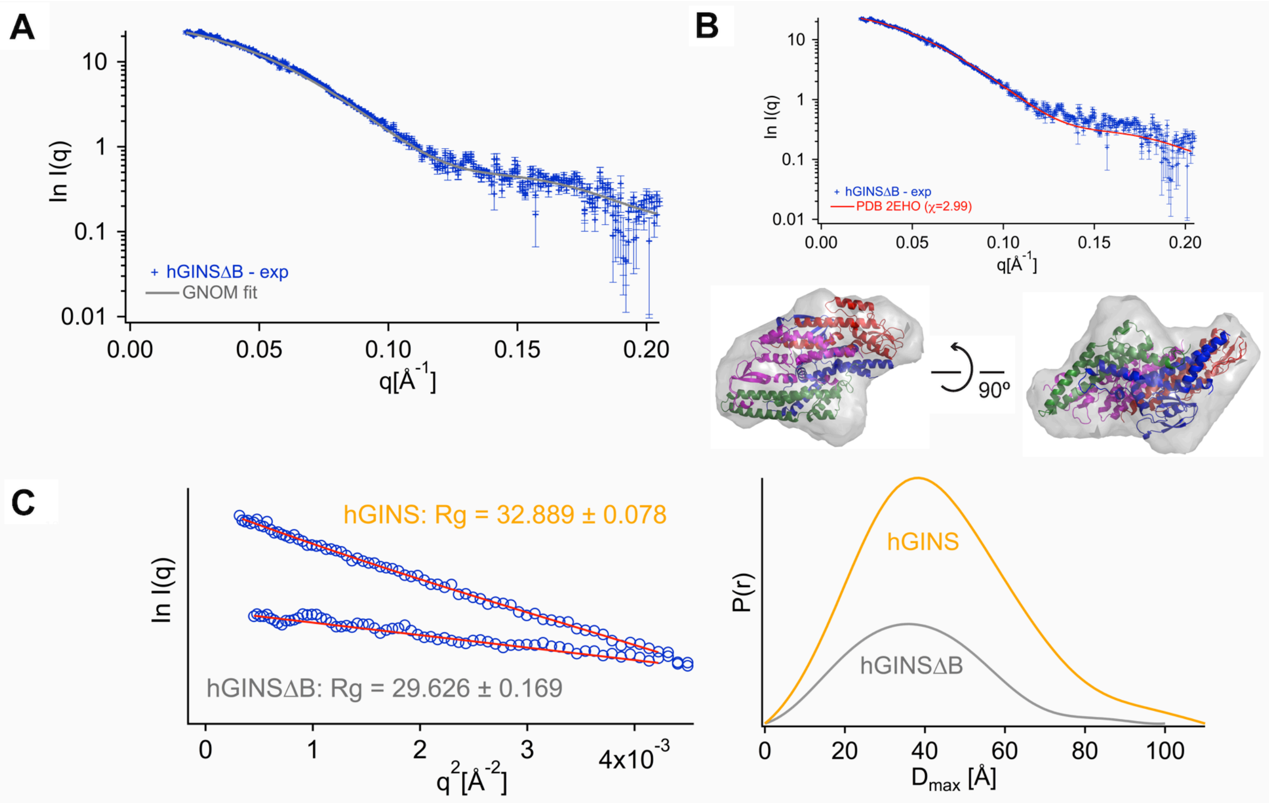


**D**

| Sample | Q range (Å⁻¹) | I(0) | Rg (Å) Gnom | Rg (Å) Igor | $D_{MAX}$ (Å) | Volume (Å) | MW (KDa) | Monomer % |
|---|---|---|---|---|---|---|---|---|
| hGINS | 0.014 – 0.22 | 0.067 | 33.69 ± 0.41 | 32.889 ± 0.078 | 110 | 193700 | 124 ± 23 | 100 |
| hGINSΔB | 0.023 – 0.20 | 0.026 | 30.02 ± 0.63 | 29.626 ± 0.169 | 100 | 105200 | 84 ± 13 | 100 |

**Comparison of the full-length and Psf1ΔB hGINS complexes by SAXS. A.** GNOM fit (grey line) of the SAXS experimental Psf1ΔB hGINS profile (blue crosses) with standard deviation. **B.** Fit of the theoretical scattering curve (red line) calculated from the crystal structure with PDB ID: 2EHO (cartoon) to the experimental profile (blue crosses) and to the final envelope (grey surface) of Psf1ΔB hGINS. **C.** Guinier's fits with experimental Rg values and evaluation of the P(r) functions with Dmax values for both hGINS (yellow) and Psf1ΔB hGINS (grey). **D.** Table summarizing the main structural parameters for both samples obtained using ATSAS software.

**Supplementary Figure S5.**

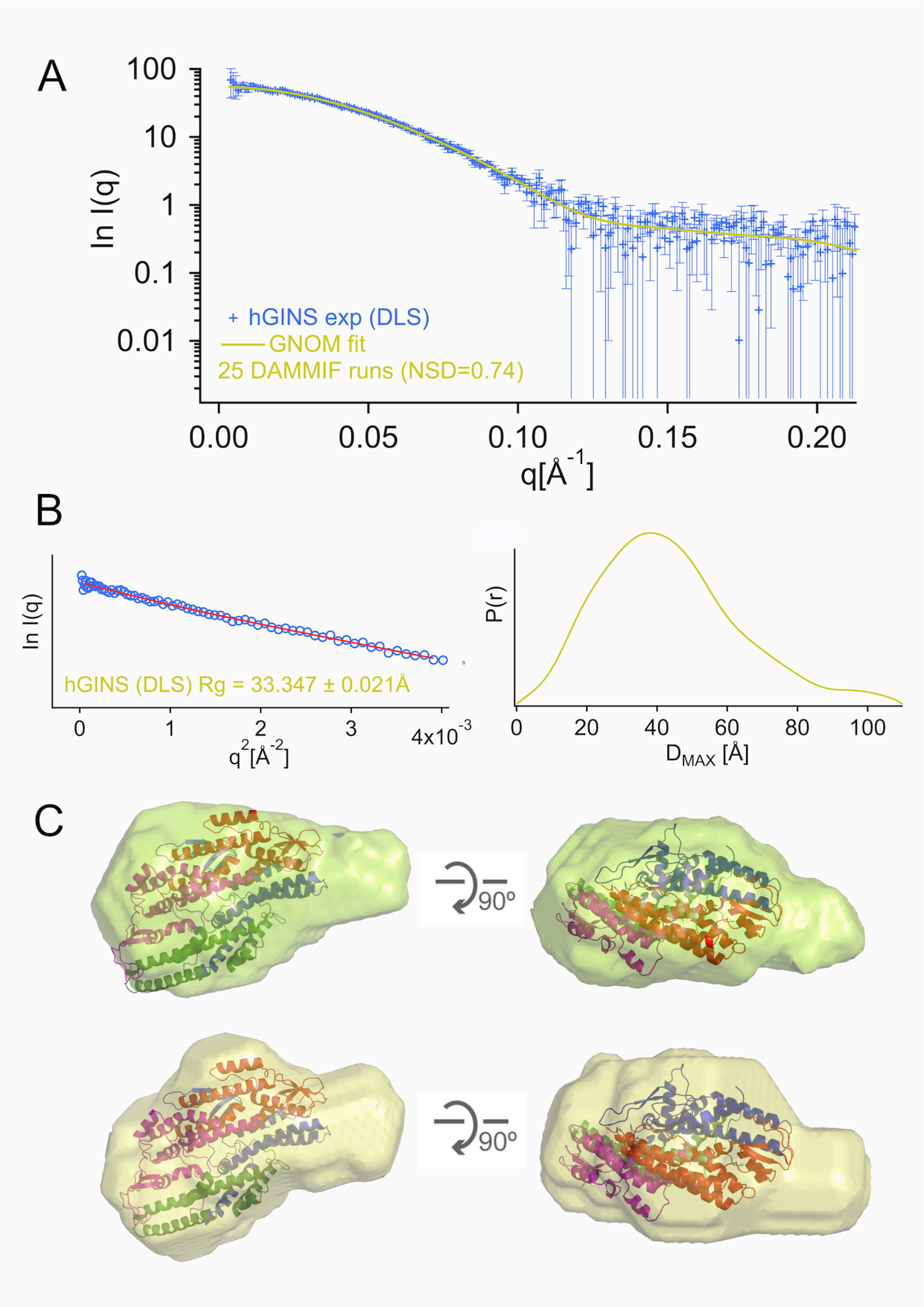


**In-line Size Exclusion Chromatography (SEC) coupled SAXS data collection. A.** Experimental SAXS profile (blue crosses) compared with the theoretical scattering curve calculated from the ab initio model (green line). **B.** Guinier's fit with experimental Rg value and P(r) function. **C.** Comparison of final envelopes obtained from in-line SEC-SAXS data (top panel, green) or the SAXS data presented in Figure 4 (botton panel, yellow).
